# SARS-CoV-2 spike behavior in situ: a Cryo-EM images for a better understanding of the COVID-19 pandemic

**DOI:** 10.1038/s41392-020-00365-7

**Published:** 2020-10-30

**Authors:** Alaa M. Ismail, Abdo A. Elfiky

**Affiliations:** Biophysics Department, Faculty of Sciences, Cairo University, Giza, Egypt

**Keywords:** Infection, Biophysics

Most recently in Nature Journal, Ke et al. thoroughly investigate the SARS-CoV-2 spike in situ by using cryo-electron microscopy (Cryo-EM) to reveal its different conformations, orientations, and distribution over the virion particles.^1^

The cornerstone of any viral infection is the recognition of the viral element, usually a glycoprotein, to the host cell receptors. Knowledge of the behavior of viral glycoprotein is crucial for understanding the mode of infection and designing a vaccine against viruses. The spike glycoprotein is the critical viral element that is responsible for host cell recognition, attachment, and entry for the human coronaviruses. The trimeric spikes are the transmembrane protein that undergoes dramatic structural rearrangements for binding to its host cell receptor, the angiotensin-converting enzyme 2 (ACE2), that mediate subsequent membrane fusion and virion entry.^2^

The novel human coronavirus strain, SARS-CoV-2, has influenced people across the globe with more than 27 M confirmed cases, from which ≥890 K deaths are reported. Drug designers and vaccine developers are intriguingly studying the spike glycoprotein of SARS-CoV-2 due to its paramount importance in viral-host cell communications. The spike glycoprotein was the first SARS-CoV-2 protein to be solved experimentally (6VSB), released on the Protein Data Bank on February 26, 2020. Due to the colossal structure of the trimeric spike (1300 residues per monomer), Cryo-EM was utilized to get the 3D structures of the different states of the trimeric spike glycoprotein.^3,4^

Ke et al. infected VeroE6 and the lung cancer Calu-3 cells with SARS-CoV-2 then imaged the fixed supernatant with Cryo-EM. The virions are slightly larger in diameter in the case of Calu-3 cells (104 ± 13 nm) compared to the VeroE6 cells (91 ± 11 nm). In both systems, the trimeric spike takes two main isoforms, the prefusion (97%) and the postfusion (3%), as shown in Fig. 1.

**Fig. 1:**
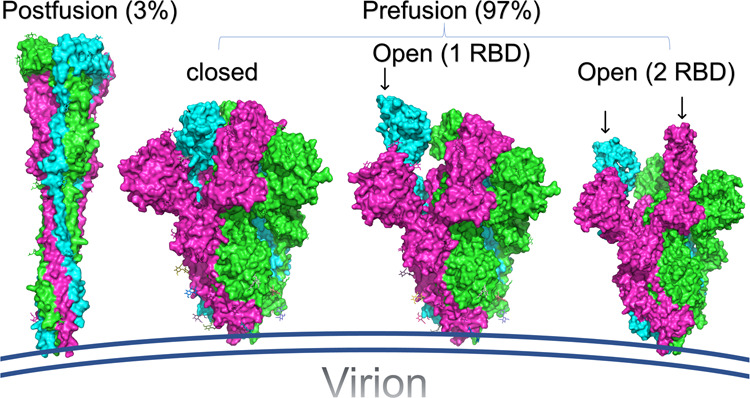
The structures of the prefusion and postfusion trimeric spike for SARS-CoV-2. The structures are represented in the colored (by chain) surfaces using PyMOL software. Small arrows show the open RBD in the prefusion subclasses. The structures for the postfusion, closed prefusion, open 1 RBD prefusion, and open 2 RBD prefusion are downloaded from the PDB with codes: 6M3W, 6VXX, 6VYB, and 6X2B, respectively

In the prefusion isoform, the spike trimers are broad and may take different conformations. Ke et al. classified the prefusion trimers according to the receptor-binding domain (RBD) orientations, into three classes; the closed, the open, and the spike trimers with mobile, but mostly closed, RBD conformations, which is characterized by a week electron density. In the open conformation, the RBD of one or two of the spike monomers is surface exposed and available for the ACE2 recognition. On the other hand, in the closed conformation of the prefusion isoform, all the three RBDs are covered by the N-terminal domain (NTD) of the spike. Ke et al. found that 31% of the prefusion spike trimers are closed, 55% have one RBD open, while 14% have two RBD available.

Accordingly, once bound to the ACE2 receptor, the prefusion spike trimer undergoes a structural transition to the postfusion isoform in which the fusion peptide and the transmembrane domains are bridged together, forming a long needle-like structure.^5^

Nonetheless, the postfusion trimers found over the free virions may be part of the defense mechanism of SARS-CoV-2. Postfusion trimers could be expressed to protect the virus from the host immune response, as it shields the prefusion trimers from the neutralizing antibodies. Thus it is essential to be considered during vaccine formulation.

The most impressive aspect is that both isoforms of the spike trimers are flexible to tilt out of the viral membrane with a tilting angle range 0^0^–90^0^, while the membrane-proximal stalk region acts as a flexible hinge. This view is critical in vaccine development as the head domain’s base and the stalk region can be accessible for neutralizing antibodies. In addition, the spike trimers are sparsely distributed over the virion membrane giving more chances for antibodies to go through the inner parts of the trimer having a lower level of glycan protection.

The morphology of the virions, as well as the spike conformation, are different in the concentrated virions compared to the unconcentrated experiments. Concentrated virions are deviated from spherical morphology, while the prefusion trimers are only either in the closed RBD (53%) or movable (week electron density) RBD (47%) conformations with no open conformation at all. Ke et al. suggested that the open RBD conformation, observed in the unconcentrated experiment, is ‘fragile,’ meaning that it can be affected by the purification procedures. This is of extreme importance since only the open conformation can bind to the ACE2 receptor over the host cell.

The in situ structures by Ke et al. for the SARS-CoV2 spike prove the legitimacy of recombinant, purified spike trimers in different research aspects and vaccination studies because they ultimately represent the various states of the spike protein on the virion particles.

Overall, the authors bring to light the details of spike protein structure on the virion surface using cryo-EM, which could be a hub in immunogene design for vaccination.

## References

[1] Ke Z (2020). Structures and distributions of SARS-CoV-2 spike proteins on intact virions. Nature.

[2] Yan R (2020). Structural basis for the recognition of SARS-CoV-2 by full-length human ACE2. Science.

[3] Wrapp D (2020). Cryo-EM structure of the 2019-nCoV spike in the prefusion conformation. Science.

[4] Walls AC (2020). Structure, function, and antigenicity of the SARS-CoV-2 spike glycoprotein. Cell.

[5] Shang J (2020). Structural basis of receptor recognition by SARS-CoV-2. Nature.

